# Early Ibrutinib Dose Modifications in CLL: A Post Hoc Analysis of the Real-World EVIdeNCE Study

**DOI:** 10.3390/cancers18061000

**Published:** 2026-03-19

**Authors:** Stefano Molica, Potito Rosario Scalzulli, Lydia Scarfò, Carla Minoia, Roberta Murru, Paolo Sportoletti, Francesco Albano, Nicola Di Renzo, Alessandro Sanna, Luca Laurenti, Massimo Massaia, Ramona Cassin, Marta Coscia, Caterina Patti, Elsa Pennese, Agostino Tafuri, Annalisa Chiarenza, Piero Galieni, Omar Perbellini, Carmine Selleri, Catello Califano, Felicetto Ferrara, Antonio Cuneo, Marco Murineddu, Gaetano Palumbo, Ilaria Scortechini, Alessandra Tedeschi, Livio Trentin, Marzia Varettoni, Fabrizio Pane, Francesco Merli, Lucia Morello, Gerardo Musuraca, Monica Tani, Adalberto Ibatici, Maria Palma, Danilo Arienti, Francesca Romana Mauro

**Affiliations:** 1 Hull York Medical School, University of Hull, Hull HU6 7RX, UK; 2 Ospedale “Casa Sollievo Della Sofferenza”, 71013 San Giovanni Rotondo, Italy; 3 School of Medicine, Università Vita Salute San Raffaele, 20132 Milan, Italy; 4 Strategic Research Program on CLL, Comprehensive Cancer Center, IRCCS Ospedale San Raffaele, 20132 Milan, Italy; 5 Hematology Unit, IRCCS Istituto Tumori “Giovanni Paolo II”, 70124 Bari, Italy; 6 Hematology and Stem Cell Transplantation Unit, Ospedale Oncologico A. Businco, ARNAS G. Brotzu, 09121 Cagliari, Italy; 7 Hematology and Clinical Immunology Section, Department of Medicine and Surgery, Center for Hemato-Oncological Research (CREO), University of Perugia, 06123 Perugia, Italy; 8 Hematology and Stem Cell Transplantation Unit, Department of Precision and Regenerative Medicine and Ionian Area (DiMePRe-J), University of Bari “Aldo Moro”, 70121 Bari, Italy; francesco.albano@uniba.it; 9 UOC Ematologia e Trapianto di Cellule Staminali, PO “Vito Fazzi”, ASL Lecce, 73100 Lecce, Italy; 10 Hematology Unit, AOU Careggi, 50134 Florence, Italy; 11 Policlinico A. Gemelli, 00168 Rome, Italy; 12 SC Ematologia, AO S. Croce e Carle, 12100 Cuneo, Italy; 13 Dipartimento di Biotecnologie Molecolari e Scienza Della Salute, Università Degli Studi di Torino, 10126 Torino, Italy; 14 Fondazione IRCCS Cà Granda Ospedale Maggiore Policlinico of Milan, 20122 Milano, Italy; 15 Department of Medicine and Surgery, University of Insubria, 21100 Varese, Italy; 16 Division of Hematology, ASST Sette Laghi, 21100 Varese, Italy; 17 UOC Oncoematologia, Ospedali Riuniti Villa Sofia-Cervello, 90146 Palermo, Italy; 18 UOC Ematologia, Dipartimento Oncologico-Ematologico, PO Santo Spirito, 65124 Pescara, Italy; 19 Ematologia, AOU Sant’Andrea, Sapienza Università di Roma, 00185 Rome, Italy; 20 UOC Ematologia, AOU Policlinico G.Rodolico-San Marco, 95123 Catania, Italy; 21 UOC Ematologia e Terapia Cellulare, Ospedale Mazzoni, 63100 Ascoli Piceno, Italy; piero.galieni@sanita.marche.it; 22 UOC Ematologia, Azienda ULSS 8 Berica, 36100 Vicenza, Italy; 23 U.O.S.D. Genetica Medica e Genomica, Ospedale S. Bortolo, Azienda ULSS N. 8 “Berica”, 36100 Vicenza, Italy; 24 Hematology and Bone Marrow Transplant Center, Department of Medicine and Surgery, University of Salerno, 84084 Salerno, Italy; 25 UOC Ematologia, PO Andrea Tortora-Pagani, 84016 Pagani, Italy; 26 AORN Antonio Cardarelli, 80131 Naples, Italy; felicettoferrara@katamail.com; 27 Hematology Unit, Università Degli Studi di Ferrara, 44121 Ferrara, Italy; cut@unife.it; 28 Ematologia, Ospedale San Francesco ASL Nuoro, 08100 Nuoro, Italy; 29 SC Ematologia, AOU Policlinico Foggia, 71122 Foggia, Italy; 30 Azienda Ospedaliero Universitaria delle Marche, 60126 Ancona, Italy; 31 Divisione di Ematologia, ASST GOM Niguarda, 20162 Milan, Italy; 32 UOC Ematologia, Dipartimento di Medicina, Università di Padova, 35122 Padua, Italy; 33 Divisione di Ematologia, Fondazione IRCCS Policlinico San Matteo, 27100 Pavia, Italy; 34 Università di Napoli Federico II, 80138 Naples, Italy; 35 Ematologia, AUSL-IRCCS Reggio Emilia, 42122 Reggio Emilia, Italy; 36 Humanitas Cancer Center, IRCCS Humanitas Research Hospital, 20089 Rozzano, Italy; 37 IRCCS Istituto Romagnolo per lo Studio dei Tumori (IRST) “Dino Amadori”, 47014 Meldola, Italy; 38 Haematology Unit, Ospedale S. Maria delle Croci, 48121 Ravenna, Italy; monica.tani@auslromagna.it; 39 IRCCS Ospedale Policlinico San Martino, 16132 Genoa, Italy; 40 Johnson & Johnson Innovative Medicine, 20126 Milan, Italy; 41 Hematology, Department of Translational and Precision Medicine, Sapienza Università di Roma, 00185 Rome, Italy; mauro@bce.uniroma1.it

**Keywords:** CLL, ibrutinib adherence, dose modifications, patient comorbidities, clinical outcomes

## Abstract

**Background/Objectives**: Ibrutinib has significantly improved outcomes in chronic lymphocytic leukemia (CLL), but evidence from real-world settings on the impact of early dose modifications and consequent relative dose intensity (RDI) maintenance on survival outcomes is limited. This study evaluated the impact of dose reductions and RDI maintenance during the first 90 days of treatment on clinical outcomes in patients with CLL receiving ibrutinib in routine clinical practice. **Methods**: A post hoc analysis of the prospective observational EVIdeNCE study (NCT03720561) was conducted, including 275 patients with CLL treated with ibrutinib. Baseline clinical and biological factors associated with early dose modifications and RDI maintenance over the first 90 days were analyzed. Cox proportional hazards models, adjusted for disease- and patient-related covariates, were applied to assess associations with overall survival (OS) and progression-free survival (PFS), using a landmark approach to control for immortal time bias. **Results**: Patients with higher comorbidity burden—indicated by higher Cumulative Illness Rating Scale scores and poorer ECOG performance status—were more likely to undergo early dose reductions. RDI declined slightly over 90 days, but most patients maintained ≥80% of their RDI. The impact of disease-risk factors appeared more clearly when assessing the relationship between 100% RDI at 90 days and PFS, with ibrutinib at 100% RDI associated with improved PFS (hazard ratio, HR 2.26, 95% confidence interval, CI: 1.23–4.15). However, after adjusting for patient characteristics (e.g., comorbidity burden and cardiovascular history), the 100% RDI rate no longer showed a statistically significant effect on PFS (HR 1.84, 95% CI: 0.93–3.63). **Conclusions**: Baseline comorbidities and functional status drive early dose modifications, but these adjustments and RDI variability do not independently impact survival outcomes, confirming the overall tolerability of ibrutinib in real-world CLL management.

## Simple Summary

This analysis evaluated treatment relative dose intensity (RDI) among patients with chronic lymphocytic leukemia receiving ibrutinib, administered either as first-line or subsequent lines of therapy, in a real-world setting. The findings indicate that a higher burden of comorbidities and poorer performance status are associated with initial dose reductions. Nonetheless, most patients maintained a high RDI rate (≥80%) during the first three months of therapy. While a 100% RDI rate at 90 days was initially associated with improved progression-free survival (PFS) in patient subsets with adverse features, this association was attenuated after adjusting for patient-specific factors such as age, comorbidities, and cardiovascular history. These results highlight the importance of individualized management of ibrutinib therapy, taking into account disease biology and comorbid conditions, to optimize clinical outcomes.

## 1. Introduction

Ibrutinib, a first-in-class covalent inhibitor of the Bruton’s tyrosine kinase (BTK), has become a key treatment option in the management of chronic lymphocytic leukemia (CLL) [1,2,3,4,5,6,7,8]. It has demonstrated sustained efficacy and has been associated with improved survival outcomes in both long-term follow-ups of either clinical trials or real-world studies [9,10,11]. Nonetheless, treatment discontinuation remains a notable challenge, predominantly due to adverse events such as atrial fibrillation and hypertension, particularly in routine clinical settings outside of controlled trials [1,2,3,4,5,6,7,8,9,10,11].

Second-generation covalent BTK inhibitors demonstrate increased selectivity and are associated with a lower incidence of cardiovascular adverse events, positioning them as a potential alternative to ibrutinib, particularly for patients with pre-existing cardiovascular comorbidities [12]. However, direct comparative studies—primarily involving patients with relapsed/refractory [R/R] CLL—have indicated that off-target effects are reduced but not completely eliminated with these second-generation agents [13,14].

This complexity in management, encompassing both first- and second-generation BTK inhibitors, underscores the significant challenges faced by healthcare providers when deciding whether to adjust the dosage of ibrutinib or to transition patients to second-generation BTK inhibitors [15].

Real-world evidence studies, including data from the EVIdeNCE (NCT03720561) and REALITY studies, suggest that the integration of ibrutinib into the treatment algorithm for CLL—initiated more than a decade ago—has enabled clinicians to develop a comprehensive, long-term understanding of its management [7,9]. These experiences underscore the critical importance of modulating ibrutinib dosage in patients with certain comorbidities to reduce the risk of discontinuation and to maximize treatment efficacy and safety [16,17,18].

Building upon data from the EVIdeNCE study—a prospective, multicenter, non-interventional investigation primarily designed to evaluate real-world utilization patterns of ibrutinib in Italy—we conducted a post hoc analysis to assess the impact of dose modifications on clinical outcomes [6]. The results indicate that dose reductions in ibrutinib are common among unselected patient populations and do not appear to compromise disease control, thereby supporting the feasibility of personalized, tolerability-driven dosing adjustments.

## 2. Materials and Methods

### 2.1. Patients

Patients diagnosed with CLL who initiated ibrutinib—either as upfront or subsequent treatment—were enrolled in the EVIdeNCE study (NCT03720561), an Italian, multicenter, observational, prospective study [6]. All enrolled patients commenced treatment with ibrutinib within routine clinical practice settings. Enrollment took place between November 2018 and October 2019 across 39 hematology centers nationwide. Exclusion criteria included participation in experimental clinical trials, as well as pregnancy or breastfeeding [6].

This post hoc analysis focuses on the impact of dose reduction or interruption of ibrutinib therapy. Patients treated at centers that employed a non-conventional starting dose for ibrutinib—specifically, three centers involving 34 patients—were excluded from this analysis. The exclusion was driven by an iterative habit among clinicians to reduce the ibrutinib dose rather than by any specific patient or baseline disease characteristic.

Patients were followed for up to 24 months from the initiation of ibrutinib therapy, regardless of treatment discontinuation. Follow-up visits were scheduled at three-month intervals during the first year and at six-month intervals thereafter. Clinical information was predominantly obtained from medical records and subsequently entered into an electronic case report form (eCRF) as the study progressed.

Baseline data collected included demographic information, detailed medical history, comorbidities—assessed using the Cumulative Illness Rating Scale (CIRS)—as well as CLL-specific characteristics (e.g., Rai stage, 17p (del) and/or TP53 aberrations) and prior treatments. During follow-up, data on treatment effectiveness, hematologic and biochemical parameters, adverse events, and vital signs were systematically documented. Additionally, any modifications to treatment, including dose reductions, interruptions, or discontinuations, were recorded.

The study adhered to the principles of the Declaration of Helsinki and to Good Clinical Practice (GCP). The protocol was approved by the Independent Ethics Committees at each participating center, and written informed consent was obtained from all participants before enrollment.

### 2.2. Dosing Regimen of Ibrutinib

Based on the initial dose received, patients were categorized into two groups: those who received the approved full dose of ibrutinib (420 mg daily) and those who received a reduced dose (less than 420 mg daily). RDI to ibrutinib therapy was assessed at specific time points—namely, 30, 60, and 90 days after therapy initiation. It was calculated for each patient as the ratio between the average daily dose received during the specified period (determined by dividing the total cumulative dose by the number of days) and the theoretical daily dose of 420 mg. For patients who interrupted or discontinued ibrutinib, a dose of 0 mg per day was assigned for each day skipped. A 100% RDI rate indicated that the patient had received the full daily dose throughout the entire period under consideration. Dosing reductions and treatment interruptions were recorded via prescription records, which served as the primary data source for exposure assessment.

### 2.3. Statistical Methods

A comparative analysis of the clinical and biological characteristics of patients who received full versus reduced doses of ibrutinib was performed. Continuous variables were reported as means with standard deviations or medians with interquartile ranges (IQR), while categorical variables were expressed as frequencies and percentages. Differences between groups were evaluated using the chi-square test or Fisher’s exact test for categorical variables, and the t-test or Wilcoxon rank-sum test for continuous variables. Predictors of reduced starting dose were evaluated using a multivariable logistic regression model. Age, sex, and additional covariates suggestively associated with starting dose in univariate analyses (e.g., comorbidity burden, particularly cardiovascular comorbidity and fitness status) were entered simultaneously into the model, and adjusted odds ratios (ORs) of reduced starting dose, with corresponding 95% confidence intervals (CIs), were reported. Genetic variables (i.e., IGHV mutation status and TP53 mutation or del(17p)) were not included due to the high proportion of missing data.

We initially assessed the impact of the starting dose of ibrutinib (full dose vs. reduced dose) on progression-free survival (PFS) and overall survival (OS). PFS was defined as the time from the initiation of ibrutinib treatment until disease progression or death without progression. The progression of the disease was evaluated according to iwCLL criteria [19]. OS was defined as the duration from treatment start to death from any cause. Subsequently, the association between PFS or OS and 100% rate of RDI to ibrutinib at three time points, i.e., 30, 60, and 90 days post-treatment initiation, was assessed. To account for immortal time bias, landmark analyses were conducted, restricting inclusion to patients who were alive and event-free at each corresponding time point.

The Kaplan–Meier method was employed to analyze PFS and OS. Hazard ratios (HRs) and their 95 CI for all-cause mortality and disease progression or death were estimated using multivariable Cox proportional hazards models. Initially, a basic model was constructed, adjusting for key demographic and treatment factors such as age, sex, and line of therapy. To further assess how patient- and disease-specific variables influence the relationship among starting dose, maintenance of RDI, and clinical outcomes, three distinct models were developed. The initial adjusted model concurrently incorporated patient-related factors (specifically, Eastern Cooperative Oncology Group Performance Status [ECOG PS], comorbidity burden as measured by the CIRS, and pre-existing cardiovascular comorbidities or cancer) and disease-related variables (notably TP53 mutation status or deletion of chromosome 17p, and Rai stage). Two additional models were separately specified to assess the independent contributions of either patient-related factors or disease-related factors to the association among starting dose, RDI, and clinical outcomes.

The proportional hazards assumption was assessed using Schoenfeld residual-based tests. No violations were detected for the all-cause mortality and disease progression/death models, including the models with the highest degree of adjustment for both starting dose (*p* = 0.813 for the all-cause mortality model, and *p* = 0.790 for the disease progression/death model) and 90-day RDI rate (*p* = 0.921 and *p* = 0.213, respectively). Missing values in covariates were handled by including a separate category in the regression models.

All statistical tests were two-sided, with a *p*-value < 0.05 indicating statistical significance. Statistical analyses were carried out using SAS software (version 9.4; SAS Institute Inc., Cary, NC, USA).

## 3. Results

### 3.1. Early Ibrutinib RDI Maintenance According to the Clinical and Biologic Characteristics of Patients

This post hoc analysis of the EVIdeNCE study included data from 275 patients with CLL who received ibrutinib as either first-line therapy (37%) or subsequent lines of treatment (63%). At the time of initial administration, ibrutinib was prescribed at the standard full dose of 420 mg in 226 patients (82.2%), while 49 patients (17.8%) received a reduced dose. Among those receiving a reduced dose, 26 patients (9.5%) were given 280 mg, and 23 patients (8.4%) received 140 mg. The distribution of patients starting on full versus reduced doses was similar regardless of whether ibrutinib was administered as initial or subsequent therapy (*p* = 0.956).

Among the 49 patients who initiated ibrutinib at a reduced starting dose, the most frequently reported reason was the physician’s preference (*n* = 37 or 75.5%). Other documented reasons included comorbidities or worsening of pre-existing conditions (*n* = 7 or 14.3%), concomitant medications (*n* = 4 or 8.2%), and other reasons (*n* = 1 or 2.0%).

To identify factors associated with dose reduction in ibrutinib, a cross-sectional analysis was conducted comparing patients who required dose adjustments to those who started with the initial full dosage. Patients in the starting dose-reduction group exhibited significantly higher median CIRS scores (median: five [range: 2–7]) compared to those in the full-dose group (median: three [range: 1–6]; *p* = 0.022). Additionally, this group demonstrated significantly worse ECOG PS scores (*p* < 0.001). Moreover, the dose-reduction cohort exhibited a higher median age (72 years; IQR, 68–79) compared with the full-dose cohort (71 years; IQR, 65–77); however, this difference did not reach statistical significance (*p* = 0.07).

Interestingly, a CIRS threshold of six—commonly employed to distinguish fit from unfit patients—did not significantly differentiate the two groups (*p* = 0.153). This finding may reflect limitations of the CIRS, originally developed during the chemo-immunotherapy era, which may be less effective for stratifying patients who are suitable candidates for full-dose ibrutinib. Similarly, the severity of individual comorbid conditions across various CIRS domains showed no notable differences between patients in the reduced-dose and full-dose groups (Table S1). The prevalence of baseline cardiovascular disorders, including hypertension, was also comparable between the two cohorts (reduced-dose vs. full-dose: 38.8% vs. 30.5%; *p* = 0.262). Furthermore, key disease characteristics, such as Rai stage (*p* = 0.256), IGHV mutation status (*p* = 0.219), and TP53 abnormalities (*p* = 0.431), were evenly distributed across patients regardless of the initial dose administered (Table 1).

In the multiple-adjusted analysis, only ECOG PS was a statistically significant predictor of reduced starting dose (OR = 8.01, 95% CI: 2.91–22.08 for ECOG PS of 2–3 vs. ECOG PS of 0–1).

### 3.2. Rate of Early Dose Reductions, Interruptions, and Disease Progression in Patients Initiated on Ibrutinib

Within the first 90 days of treatment, 20 (8.8%) of 226 patients who initiated ibrutinib at the full dose required at least one dose reduction, totaling 24 reductions. Toxicity was the leading driver (45.8% of the reduction events), followed by physician preference (25.0%) and other reasons (25.0%); one reduction (4.2%) was due to comorbidities or worsening of pre-existing conditions.

In the whole cohort, temporary interruptions (<3 months) within the first 90 days of treatment occurred in 41 patients (14.9%), accounting for 43 episodes. Toxicity accounted for 44.2% of interruptions, with other reasons comprising 41.9%; the remaining 14.0% were due to the physician’s preference (7.0%) or unspecified causes (7.0%). Early temporary interruptions were equally distributed in the full-dose group (31 of 226 or 13.7%) versus the reduced-dose group (10 of 49 or 20.4%) (*p* = 0.23). Overall, early disease progression within 90 days was observed in five patients (1.8%), distributed as four in the full-dose cohort and one in the reduced-dose cohort.

### 3.3. Impact of Early Ibrutinib 100% RDI Rate on Clinical Outcomes

**Figure 1:**
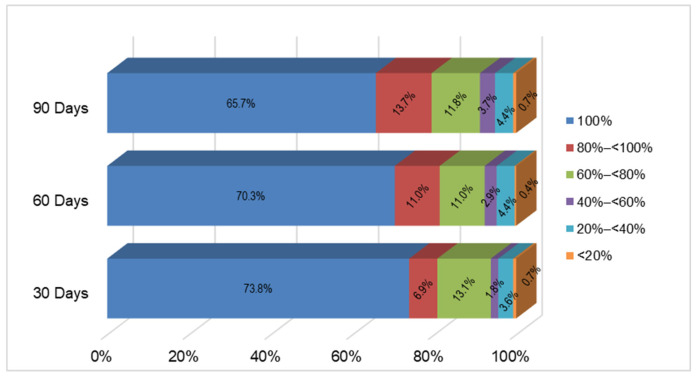
Ibrutinib relative dose intensity (RDI) rate during the first 30, 60, and 90 days of treatment. All patients had total follow-up time >30; two patients with total follow-up time between 30 and 59 days did not contribute to the RDI assessment during the first 60 days; 4 patients with total follow-up time between 30 and 89 days did not contribute to the RDI assessment during the first 90 days. The analysis included 1 patient who discontinued permanently within the first 30 days, and 11 patients who discontinued between 60 and 90 days.

The 100% RDI rate at 30, 60, and 90 days post-treatment initiation was observed in 73.8%, 70.3%, and 65.7% of patients, respectively (Figure 1). Despite a gradual decline, 79.4% of patients had a ≥80% RDI rate at the 90-day time point.

**Figure 2:**
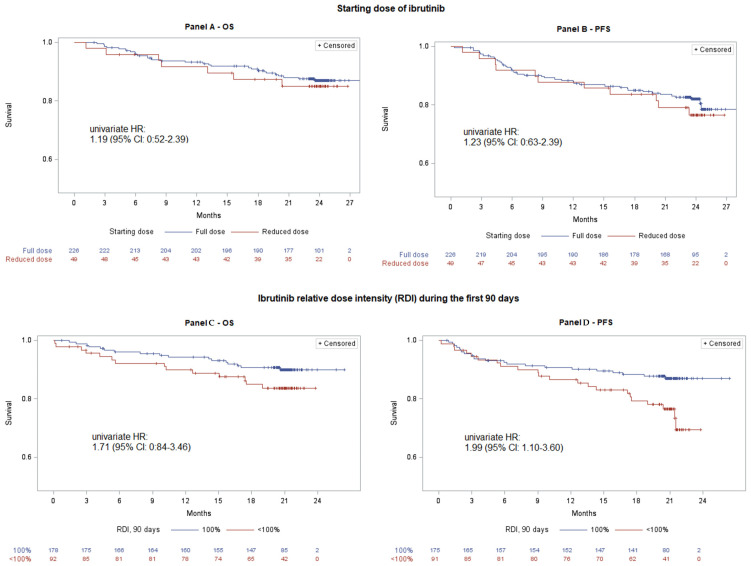
Kaplan–Meier curves, and univariate hazard ratios (HR) with corresponding 95% confidence intervals (CI), of overall survival (OS) and progression-free survival (PFS) according to the ibrutinib starting dose (Panels (**A**,**B**)) and ibrutinib 100% RDI rate during the first 90 days of treatment (Panels (**C**,**D**)). A 90-day landmark analysis was used for 90-day RDI. For the PFS analyses, events were defined as disease progression or death without progression.

Subsequently, we examined the potential association between the RDI to initial ibrutinib dose and clinical outcomes. In univariate analyses, OS and PFS did not differ significantly between patients who commenced treatment at reduced doses versus those who started at the full dose. Specifically, the univariate HR for all-cause mortality was 1.19 (95% CI, 0.52–2.39), and for disease progression/death was 1.23 (95% CI, 0.63–2.39) (Figure 2, Panels A and B).

After stratifying patients by 100% RDI at 30, 60, and 90 days, we assessed the association between sustained RDI and clinical outcomes at each time point. Overall, the analysis demonstrated an increasing-strength association between RDI maintenance and favorable outcomes over time (Figure S1, Panels A to D; Figure 2, Panels C and D). The most significant associations were observed at the 90-day mark, where a full 100% RDI rate was associated with improved OS and PFS (Figure 2, Panels C and D). Specifically, at 90 days, the HRs from univariate analysis were 1.71 (95% CI: 0.84–3.46) for OS and 1.99 (95% CI: 1.10–3.60) for PFS.

**Figure 3:**
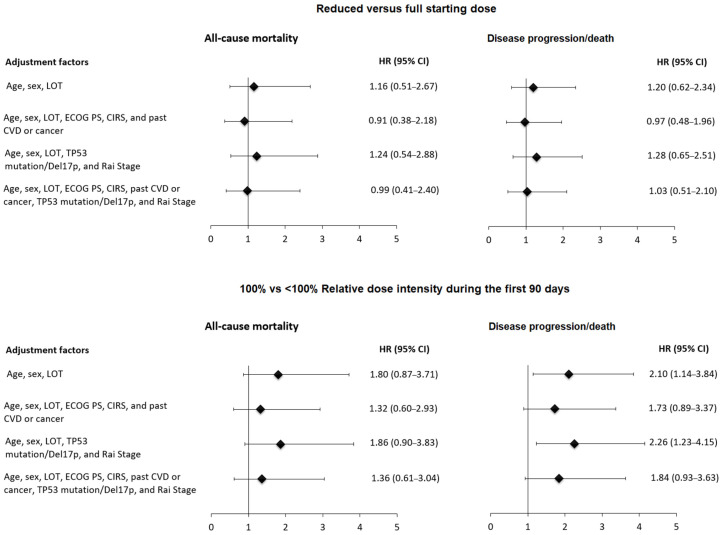
Hazard ratios (HR) and corresponding 95% confidence intervals (CI) of all-cause mortality and disease progression/death without progression according to the starting dose of ibrutinib and ibrutinib 100% RDI rate during the first 90 days of treatment, according to Cox models with different adjustment factors. For RDI, a 90-day landmark analysis was conducted. Abbreviations: LOT, line of therapy; CIRS, Cumulative Illness Rating Scale; CVD, cardiovascular diseases; ECOG PS, Eastern Cooperative Oncology Group performance status.

To account for potential confounding factors, multivariable analyses were performed using models that included either a combined set of patient- and disease-related characteristics or models primarily focused on patient- or disease-specific variables. The analysis of the impact of ibrutinib 100% RDI rate at therapy initiation (Figure 3, Panels A and B), as well as at 30 and 60 days (Table S2), did not demonstrate a statistically significant association with mortality or disease progression/death.

When the analysis was extended to 90 days, a marginal association between 100% RDI rate and OS was observed within the model, including mainly disease aggressiveness variables such as presence of TP53 aberrations, advanced Rai stage, and the number of prior treatment lines, with a HR of 1.86 (95% CI: 0.90–3.83) (Figure 3). The influence of driven disease risk factors was more evident when examining the relationship between 100% RDI rate at 90 days and PFS (HR 2.26, 95% CI: 1.23–4.15) (Figure 3). However, after adjusting also for patient characteristics such as comorbidity burden and cardiovascular history, ibrutinib 100% RDI rate did not demonstrate a statistically significant impact on PFS (HR 1.84, 95% CI: 0.93–3.63) (Figure 3).

## 4. Discussion

This study offers critical insights into ibrutinib-based CLL management in routine practice, underscoring the importance of personalized dosing strategies. Our findings reveal that clinical factors—such as age, performance status, and comorbidity burden—predominate over disease-specific biology in driving dose modifications.

Notably, cardiovascular comorbidities were equally common among patients receiving full and reduced doses, suggesting that well-controlled cardiovascular disease should not automatically contraindicate standard dosing, provided appropriate management. These results advocate for individualized dose adjustments based on tolerability rather than comorbidities alone [18].

Our longitudinal analysis demonstrated that 100% RDI rate at 30 and 60 days lacked predictive value for long-term outcomes. Conversely, 100% RDI rate at 90 days was a significant predictor of PFS in patient subsets with adverse features—particularly those with TP53 aberrations and/or del(17p), or advanced Rai stage—highlighting the importance of sustained 100% RDI rate in biologically adverse cohorts. In contrast, 100% RDI maintenance at this time point was less predictive among elderly patients with multiple comorbidities, underscoring the necessity for individualized management and realistic prognostic expectations. Evidence from clinical trials and real-world studies demonstrates that dose reductions enhance tolerability without compromising efficacy. A pooled analysis of over 1200 patients showed lower doses reduce cardiac adverse events, notably atrial fibrillation, while maintaining therapeutic benefit [16]. Additionally, real-world data indicate that dose reductions after adverse events are associated with longer time to next treatment and decreased healthcare utilization, even among high cardiovascular risk patients, when analyzed with rigorous statistical methods to minimize bias [18,20].

Pharmacological studies corroborate clinical observations, indicating that substantially lower doses of ibrutinib than currently approved can achieve comparable target engagement [21]. This issue extends beyond ibrutinib, revealing a broader limitation in targeted therapy development: approvals often rely on maximum tolerated dose paradigms with insufficient dose optimization [22]. In response, the FDA launched Project Optimus [23] to reform oncology dose-finding, aiming for patient-centered dosing that balances benefit with safety and tolerability.

While second-generation BTK inhibitors such as acalabrutinib and zanubrutinib [18,24,25] generally demonstrate improved safety profiles, data on the impact of dose reductions on their efficacy remain limited. Due to their distinct pharmacologic selectivity and pharmacokinetics, it remains uncertain whether dose modifications would produce effects comparable to those observed with ibrutinib [26,27].

Phase 3 GLOW and phase 2 CAPTIVATE support fixed-duration ibrutinib + venetoclax (I + V) for CLL [28,29,30]. The CLL17 trial shows I + V is non-inferior to continuous ibrutinib with better safety in treatment-naïve, all-comers CLL [31]. Whether the dose reductions in ibrutinib for patients on continuous treatment also successfully apply to those who experience adverse events while receiving I + V combination therapy is not yet available. The ongoing randomized TAILOR trial (NCT05963074) is designed to assess the safety, tolerability, and long-term outcomes of patients receiving varying doses of ibrutinib in both continuous and fixed-duration treatment settings. The results of this study aim to clarify whether reducing the dosage of ibrutinib can enhance tolerability while maintaining its effectiveness. Preliminary target occupancy data suggest that, in the context of continuous therapy, reduced doses of ibrutinib achieve approximately 99% occupancy comparable to that achieved with the full dose [32].

This post hoc analysis is subject to several limitations. First, the subgroup analyses were underpowered, thereby reducing the precision of the effect estimates, and RDI was evaluated within the initial 90-day period, which may not accurately reflect long-term treatment behaviors. This time point aligns with the recommended window for capturing meaningful adherence-related effects, as identified by Barr et al., who found an 8-week period to be most appropriate [33].

Moreover, in our real-world context, the rationale for initial dose reductions was not always specified, making it difficult to discern whether such modifications were proactive or reactive in nature. A further limitation is the presence of missing data for some variables, particularly genetic markers [i.e., TP53/17p(del)], a common issue in real-world studies where molecular testing is not uniformly performed or recorded. In addition, although adjustments were made for several key clinical covariates, residual confounding cannot be excluded. In particular, we were not able to account for socioeconomic status, cognitive function, and institutional prescribing patterns. Finally, results of subgroup analyses are exploratory and hypothesis-generating, as incomplete data on key stratification factors limit the robustness of these findings.

Nonetheless, the findings of this study suggest that clinical judgment—rather than disease biology—should serve as the primary consideration when determining ibrutinib dose modifications, as such adjustments do not appear to compromise therapeutic efficacy in real-world settings. Furthermore, the results suggest that cardiovascular comorbidities, when effectively managed, should not be regarded as absolute contraindications to optimal ibrutinib dosing [34]. Finally, the observation that about 80% of the patients had a high RDI rate (near 80%) during the first three months of therapy in this cohort further supports the overall tolerability of ibrutinib across a heterogeneous patient population.

## 5. Conclusions

The findings of this study showed that baseline comorbidities and functional status are the main drivers of early dose modifications in patients with CLL treated with ibrutinib. However, these adjustments are not consistently associated with overall or progression-free survival, confirming the robustness of ibrutinib efficacy across diverse patient subgroups. The substantial proportion of patients with a high RDI rate (≥80%) during the initial treatment phase further supports the favorable tolerability of ibrutinib in routine clinical practice. Collectively, these results emphasize the importance of individualized management strategies that integrate both disease biology and patient comorbidity profiles to optimize therapeutic outcomes and ensure long-term treatment success in real-world CLL care.

## Figures and Tables

**Table 1:** Association of patients’ baseline characteristics ^ with ibrutinib starting dose.

	Reduced Dose (*n* = 49)	Full Dose (*n* = 226)	*p*-Value *		OR of Reduced Dose (95% CI)
Age at ibrutinib start, median (q1–q3)	72 (68–79)	71 (65–77)	0.076		
<65	9 (18.4)	50 (22.1)		}	Ref.
65–69	5 (10.2)	43 (19.0)			
≥70	35 (71.4)	133 (58.9)	0.212		1.72 (0.83–3.58) ^¶^
Sex					
Male	32 (65.3)	142 (62.8)			Ref.
Female	17 (34.7)	84 (37.2)	0.745		0.86 (0.43–1.72)
Treatment line					
First-line	18 (36.7)	84 (37.2)			
≥Second-line	31 (63.3)	142 (62.8)	0.956		
Rai stage					
0–II	26 (54.2)	97 (45.1)			Ref.
III–IV	22 (45.8)	118 (54.9)	0.256		0.54 (0.27–1.09)
Unknown/not evaluated	1	11			
Binet stage					
A/B	22 (45.8)	96 (47.1)			
C	26 (54.2)	108 (52.9)	0.878		
Unknown/not evaluated	1	22			
CIRS, median (q1–q3)	5 (2–7)	3 (1–6)	0.022		
<6	21 (60.0)	139 (72.0)			Ref.
≥6	14 (40.0)	54 (28.0)	0.153		1.15 (0.50–2.67)
Unknown/not evaluated	14	33			
ECOG PS grade					
0	13 (34.2)	131 (66.8)		}	Ref.
1	14 (36.8)	53 (27.0)			
2	5 (13.2)	10 (5.1)		}	8.01 (2.91–22.08) ^†^
3	6 (15.8)	2 (1.0)	<0.001		
Unknown/not evaluated	11	30			
Comorbidities/relevant medical history	31 (63.3)	136 (60.2)	0.688		
Prior malignancy	6 (12.2)	15 (6.6)	0.230		
Prior bleeding events	0 (0)	1 (0.44)			
History of treated AIHA	3 (6.1)	7 (3.1)	0.391		
History of uncontrolled or symptomatic arrhythmias, AF, CHF, or MI	3 (6.1)	8 (3.5)	0.420		
History of other significant CVD	18 (36.7)	64 (28.3)	0.243		
History of significant respiratory disease	4 (6.2)	12 (5.3)	0.498		
History of systemic infection or grade 3–4 infection	0 (0)	4 (1.8)			
History of hepatitis B or C infection	4 (8.2)	24 (10.6)	0.796		
Concomitant renal dysfunction	1 (2.0)	4 (1.8)	0.999		
Diabetes	5 (10.2)	17 (7.5)	0.561		
Other	13 (26.5)	63 (27.9)	0.849		
All CVD ^¶^	19 (38.8)	69 (30.5)	0.262		0.94 (0.46–1.93)
Unmutated IGHV	16/20 (80.0)	70/106 (66.0)	0.219		
TP53 mutation/del(17p)					
absent	11 (42.3)	67 (50.8)			
present	15 (57.7)	65 (49.2)	0.431		
Unknown/not evaluated ^¥^	23	94			

Abbreviations: AF, atrial fibrillation; AIHA, autoimmune hemolytic anemia; CHF, congestive heart failure; CI, confidence interval; CVD, cardiovascular diseases; ECOG PS, Eastern Cooperative Oncology Group performance status; MI, myocardial infarction; OR, odds ratio; Ref., reference category. ^ Variables are presented as absolute numbers with percentages, unless otherwise specified; percentages are calculated among non-missing values for variables including an “unknown/not evaluated” category. * From chi-square or Fisher’s exact test for categorical variables; from the Wilcoxon test for quantitative variables. History of uncontrolled or symptomatic arrhythmias, AF, CHF, or MI, or history of other significant CVD (including hypertension). ^¥^ This category also included patients with unmutated TP53 and no available data for del17p, and vice versa. ^¶^ OR for age ≥ 70 versus <70. ^†^ OR for ECOG-PS of 2–3 versus 0–1.

## Data Availability

The datasets generated and/or analyzed during the current study are available from the corresponding author upon reasonable request. Requests for the data underlying this publication require a detailed, hypothesis-driven statistical analysis plan that is collaboratively developed by the requester and company subject-matter experts.

## Supplementary Materials

The following supporting information can be downloaded at: https://www.mdpi.com/article/10.3390/cancers18061000/s1, Figure S1: 30-day and 60-day landmark Kaplan–Meier curves of overall survival (OS) and progression-free survival (PFS) according to ibrutinib relative dose intensity (RDI) during the first 30 (Panel A and B) and 60 days of treatment (Panel C and D) in the EVIdeNCE study. For the PFS analyses, events were defined as disease progression or death without progression. Table S1: Cumulative Illness Rating Scale (CIRS) domains according to ibrutinib starting dose in the EVIdeNCE study. Table S2: Hazard ratios (HRs) with various degrees of adjustment, with corresponding 95% confidence intervals (CIs), of overall mortality and disease progression/death according to ibrutinib starting dose and ibrutinib relative dose intensity (RDI) during the first 30, 60, and 90 days of treatment in the EVIdeNCE study. For RDI, 30-, 60-, and 90-day landmark analyses were applied.
