# Supplementary material for: Early Ibrutinib Dose Modifications in CLL: A Post Hoc Analysis of the Real-World EVIdeNCE Study
Source: Cancers (Basel). 2026 Mar 19;18(6):1000. doi: 10.3390/cancers18061000 (PMC13024652; doi:10.3390/cancers18061000)
Supplement: Supplementary file 1 [file cancers-18-01000-s001.zip › cancers-4115163-supplementary.pdf]

Early Ibrutinib Dose Modifications in CLL: A Post Hoc Analysis of the Real-World EVIdeNCE Study

Supplementary Materials

Ibrutinib relative dose intensity (RDI) during the first 30 days

Panel A—OS

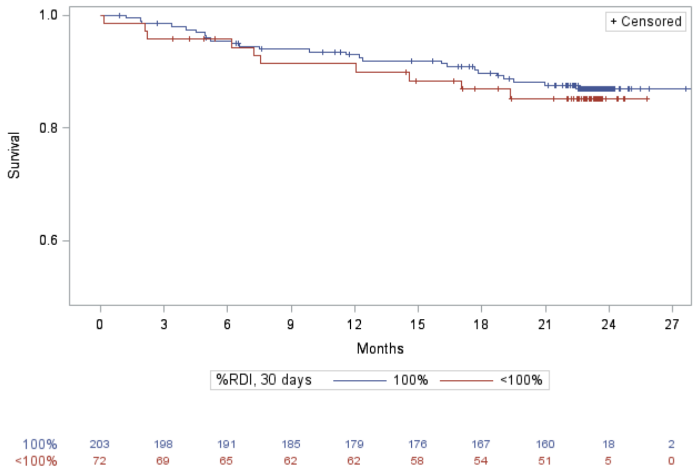

Panel B—PFS

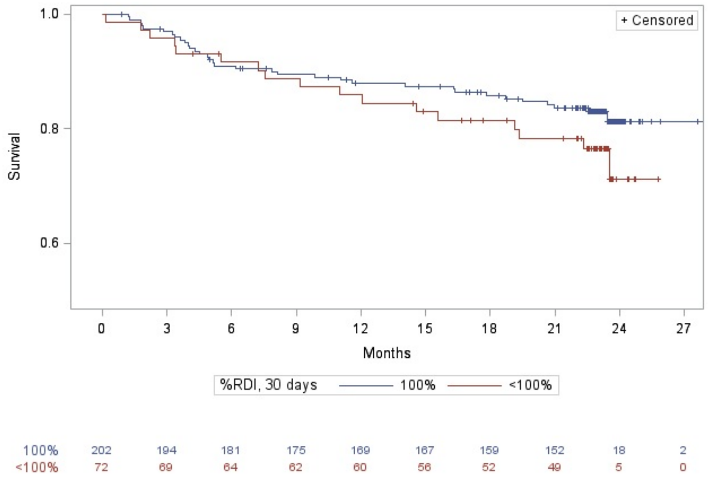

Ibrutinib relative dose intensity (RDI) during the first 60 days

Panel C—OS

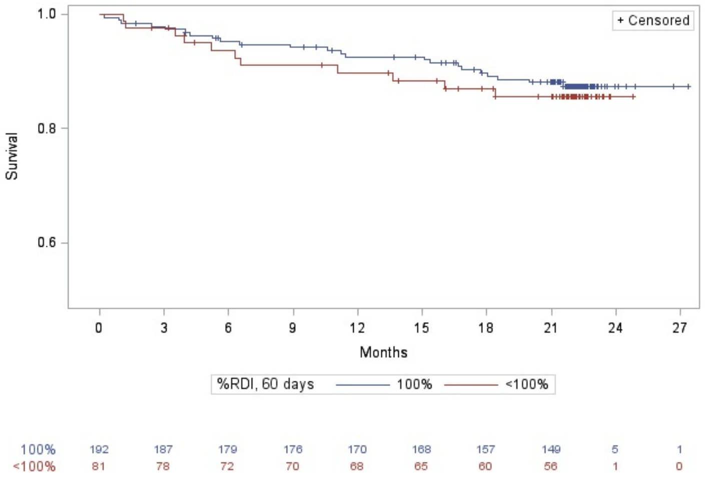

Panel D—PFS

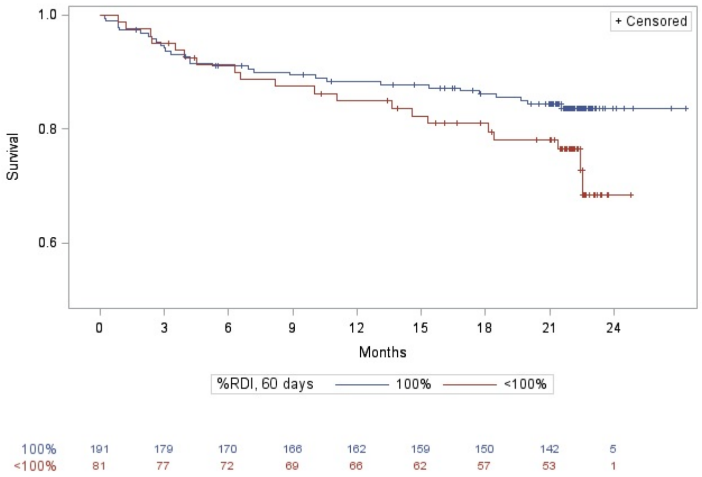

Figure S1. 30-day and 60-day landmark Kaplan–Meier curves of overall survival (OS) and progression-free survival (PFS) according to ibrutinib relative dose intensity (RDI) during the first 30 (Panel A and B) and 60 days of treatment (Panel C and D) in the EVIdeNCE study. For the PFS analyses, events were defined as disease progression or death without progression.

Table S1. Cumulative Illness Rating Scale (CIRS) domains according to ibrutinib starting dose in the EVIdeNCE study.

	**Reduced dose (n=35 ^)**	**Full dose (n=193 ^)**	*p-value**
CIRS Cardiovascular System			

0-1	29 (82.9)	173 (89.6)	
2-4	6 (17.1)	20 (10.4)	*0.251*
CIRS Vascular System			
0-1	31 (88.6)	142 (73.6)	
2-4	4 (11.4)	51 (26.4)	*0.056*
CIRS Hematological System			
0-1	29 (82.9)	166 (76.2)	
2-4	6 (17.1)	27 (14.0)	*0.626*
CIRS Respiratory System			
0-1	33 (94.3)	184 (95.3)	
2-4	2 (5.7)	9 (4.7)	*0.679*
CIRS Genitourinary System			
0-1	34 (97.1)	188 (97.4)	
2-4	1 (2.9)	5 (2.6)	*0.999*
CIRS Eye, Ear, Nose, and Throat			
0-1	34 (97.1)	190 (98.5)	
2-4	1 (2.9)	3 (1.6)	*0.489*
CIRS Hepatic System			
0-1	32 (91.4)	179 (92.8)	
2-4	3 (8.6)	14 (7.2)	*0.730*
CIRS Renal System			
0-1	33 (94.3)	187 (96.9)	
2-4	2 (5.7)	6 (3.1)	*0.354*
CIRS Gastrointestinal System			
0-1	33 (94.3)	172 (89.1)	
2-4	2 (5.7)	21 (10.9)	*0.543*
CIRS Musculoskeletal and Integumentary System			
0-1	35 (100.0)	183 (94.8)	
2-4	0 (0.0)	10 (5.2)	*0.367*
CIRS Neurological System			
0-1	33 (94.3)	190 (98.5)	
2-4	2 (5.7)	3 (1.5)	*0.170*
CIRS Endocrine/Metabolic System			
0-1	32 (91.4)	162 (83.9)	
2-4	3 (8.6)	31 (16.1)	*0.252*
CIRS Psychiatric System			
0-1	34 (97.1)	190 (98.5)	
2-4	1 (2.9)	3 (1.5)	*0.489*
CIRS Other System			
0-1	35 (100.0)	188 (97.4)	
2-4	0 (0.0)	5 (2.6)	*0.999*

Legend for CIRS levels: 0 = no problem affecting this system, 1 = current mild problem or past significant problem, 2 = moderate disability or morbidity and/or requires first-line therapy, 3 = severe problem and/or constant and significant disability and/or hard-to-control chronic problems, 4 = extremely severe problem and/or immediate treatment required and/or organ failure and/or severe function impairment. ^ This analysis was conducted in the subsample of 35 and 193 patients who started, respectively, reduced and full dose ibrutinib, and for whom baseline domain-level CIRS data were available.
* p-values from chi-square or Fisher's exact test.

Table S2. Hazard ratios (HRs) with various degrees of adjustment, with corresponding 95% confidence intervals (CIs), of overall mortality and disease progression/death* according to ibrutinib starting dose and ibrutinib relative dose intensity (RDI) during the first 30, 60, and 90 days of treatment in the EVIdeNCE study. For RDI, 30-, 60-, and 90-day landmark analyses were applied.

	All-cause mortality					Disease progression/death*					
	n. events (%)	HR# (95% CI)	HR¥ (95% CI)	HR¶ (95% CI)	HR° (95% CI)		n. events (%)	HR# (95% CI)	HR¥ (95% CI)	HR¶ (95% CI)	HR° (95% CI)
**Ibrutinib starting dose**						**Ibrutinib starting dose**					
**Full (n=226)**	28 (12.4)	Ref.	Ref.	Ref.	Ref.	**Full (n=226)**	41 (18.1)	Ref.	Ref.	Ref.	Ref.
**Reduced (n=49)**	7 (14.3)	1.16 (0.51-2.67)	0.91 (0.38-2.18)	1.24 (0.54-2.88)	0.99 (0.41-2.40)	**Reduced (n=49)**	11 (22.5)	1.20 (0.62-2.34)	0.97 (0.48-1.96)	1.28 (0.65-2.51)	1.03 (0.51-2.10)
**Ibrutinib RDI**						**Ibrutinib RDI**					
**30 days**						**30 days**					
**100% (n=203)**	25 (12.3)	Ref.	Ref.	Ref.	Ref.	**100% (n=202)**	34 (16.8)	Ref.	Ref.	Ref.	Ref.
**<100% (n=72)**	10 (13.9)	1.15 (0.55-2.40)	0.82 (0.37-1.82)	1.16 (0.55-2.44)	0.83 (0.37-1.86)	**<100% (n=72)**	17 (23.6)	1.38 (0.77-2.49)	1.11 (0.59-2.08)	1.48 (0.82-2.67)	1.18 (0.62-2.23)
**60 days**						**60 days**					
**100% (n=192)**	23 (12.0)	Ref.	Ref.	Ref.	Ref.	**100% (n=191)**	30 (15.7)	Ref.	Ref.	Ref.	Ref.
**<100% (n=81)**	11 (13.6)	1.18 (0.57-2.43)	0.83 (0.38-1.85)	1.19 (0.58-2.46)	0.84 (0.37-1.86)	**<100% (n=81)**	20 (24.7)	1.57 (0.89-2.78)	1.27 (0.68-2.38)	1.65 (0.92-2.93)	1.30 (0.68-2.46)
**90 days**						**90 days**					
**100% (n=178)**	17 (9.6)	Ref.	Ref.	Ref.	Ref.	**100% (n=175)**	22 (12.6)	Ref.	Ref.	Ref.	Ref.
**<100% (n=92)**	14 (15.2)	1.80 (0.87-3.71)	1.32 (0.60-2.93)	1.86 (0.90-3.83)	1.36 (0.61-3.04)	**<100% (n=91)**	22 (24.2)	2.10 (1.14-3.84)	1.73 (0.89-3.37)	2.26 (1.23-4.15)	1.84 (0.93-3.63)

* Progression or death without progression. # Adjusted for age, sex, and line of therapy. ¥ Adjusted for age, sex, line of therapy, Eastern Cooperative Oncology Group performance status (ECOG PS), Cumulative Illness Rating Scale (CIRS), and history of cardiovascular disease or cancer. ¶ Adjusted for age, sex, line of therapy, TP53 mutation and/or deletion of chromosome 17p, and Rai stage. ° Adjusted for age, sex, line of therapy, ECOG performance status, CIRS, history of cardiovascular disease or cancer, TP53 mutation and/or deletion of chromosome 17p, and Rai stage.
